# Development of phospholipon®90H complex nanocarrier with enhanced oral bioavailability and anti-inflammatory potential of genistein

**DOI:** 10.1080/10717544.2022.2162158

**Published:** 2023-01-01

**Authors:** Vaishnavi S. Shete, Darshan R. Telange, Nilesh M. Mahajan, Anil M. Pethe, Debarshi K. Mahapatra

**Affiliations:** a Datta Meghe College of Pharmacy, Datta Meghe Institute of Medical Sciences (Deemed to be University), Wardha, Maharashtra, India; b Dadasaheb Balpande College of Pharmacy, Nagpur, Maharashtra, India

**Keywords:** Genistein, Phospholipon®90H, solubility, dissolution, anti-inflammatory activity

## Abstract

Genistein (GEN), an isoflavonoid, offers multifunctional biological activities. However, its poor oral bioavailability, aqueous solubility, extensive metabolism, and short half-life restricted its clinical use. Therefore, the Phospholipon^®^90H complex of genistein (GPLC) was prepared to enhance its biopharmaceutical properties and anti-inflammatory activity. GPLC was characterized by employing particle size and zeta potential, Fourier transforms infrared spectrophotometry, differential scanning calorimetry, powder x-ray diffractometry, proton nuclear magnetic resonance, aqueous solubility, *in vitro* dissolution, ex vivo permeation, oral bioavailability and in vivo anti-inflammatory activity. The complex showed high entrapment of GEN (∼97.88% w/w) within the Phospholipon^®^90H matrix. Particle size and zeta potential studies confirmed the small particle size with the modest stability of GPLC. The characterization analysis supported the formation of GPLC through the participation of hydrogen bonding between GEN and Phospholipon^®^90H. GPLC significantly enhanced the aqueous solubility (∼2-fold) compared to GEN. Dissolution studies revealed that GPLC drastically improved the GEN dissolution rate compared to GEN. Likewise, the complex improved the permeation rate across the membrane compared to GEN. GPLC formulation significantly enhanced the oral bioavailability of GEN via improving its Cmax, tmax, AUC, half-life and mean residence time within the blood circulation compared to GEN. The GPLC (∼20 mg/kg, p.o.) remarkably inhibited the increase in paw edema up to 5 h, compared to GEN and diclofenac. Results suggest that the Phospholipon^®^90 complex is a superior and promising carrier for enhancing the biopharmaceutical parameters of GEN and other bioactive with similar properties.

## Graphical Abstract

**Figure:**
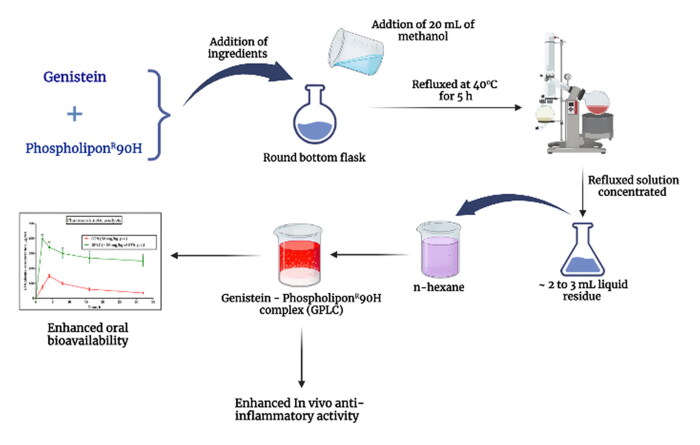

## 1. Introduction

Genistein (GEN) (IUPAC name: 4′, 5, 7-trihydroxy isoflavone or 5, 7-dihydroxy-3-(4-hydroxyphenyl) chromen-4-one), an isoflavonoid compound, shows high availability in soy-based products such as soy cheese and soy drinks (Spagnuolo et al. 2015). GEN shows molecular weight and hydroxylation pattern similarity with estradiol; therefore, GEN is also referred to as phytoestrogen (Pavese et al., 2010). GEN produces several biological effects, including antioxidant (Lopes De Azambuja et al., 2015, Braber et al., 2018), anti-inflammatory (Cai et al., 2022, Chang et al., 2015), antidiabetic (Li et al., 2020, Dkhar et al., 2018), and anti-cancer (Hussein et al., 2022, Pavese et al., 2010). Despite its positive effects, GEN demonstrates low oral bioavailability (∼23%) and could be attributed to poor aqueous solubility (∼5.3 µM), lipophilic compound (Log *P* ∼3.04) and extensive metabolism of GEN to various metabolites, including dihydrogenistein, dihydrodaidzein, 6′-hydroxy-O-desmethylangolensin, 4-ethylphenol, glucuronide and sulfate conjugates (Kobayashi et al., 2013; Schoefer et al., 2002; Tamura et al., 2007; Wu et al., 2010). Additionally, the average elimination half-life of GEN (∼3.6 h) may also find the reason for GEN poor oral bioavailability. Therefore, additional investigations are warranted to modify and improve the physico-chemical properties of GEN using a suitable nanocarrier and to achieve maximum therapeutic efficacy in the target site.

The formulation scientists have adopted many strategies to overcome GEN-described limitations. Including surface modified phytosomes (Komeil et al., 2022), metal-organic framework (Botet-Carreras et al., 2021), transmucosal solid lipid nanoparticles (Obinu et al., 2021), microemulsion (Vu et al., 2021), lactalbumin nanoparticles (Dev et al., 2021), phytosomes (Komeil et al., 2021), zein/carboxymethyl chitosan nanoparticles (Xiao et al., 2020), folic acid-conjugated chitosan nanoparticles (Cai et al., 2017) and chitosan/Eudragit nanoparticles ((Patel et al., 2015). After carefully reviewing these reports, we noticed that the authors only addressed the anti-cancer potential of these nanoformulations without analysis of GEN solubility, permeability, oral bioavailability, and anti-inflammatory activity. Therefore, based on the earlier shortcomings of the studies, we have reported a novel and promising genistein – Phospholipon^®^90H complex (GPLC) with enhanced aqueous solubility, permeability, oral bioavailability, and anti-inflammatory potential of GEN.

Phospholipids complex are lipid biocompatible molecular adducts. The complex, in association with aqueous media, rapidly transforms into spherical shape micelles. Phospholipids complex nanocarriers have shown incredible therapeutic potential among other nanocarriers because of the prevention of drug first-pass metabolism, enhancement of therapeutic drug efficacy, sustained and controlled delivery, higher encapsulation efficiency, and physical and chemical stability. Apart from these, it facilitates the transportation of API (Active Pharmaceutical Ingredients) across the intestinal barriers via adopted amphiphilic characteristics (Küllenberg et al., 2012; Li et al., 2015; Lu et al., 2019). A group of studies carried out by authors also provide evidence that phospholipids complex drastically improved the biopharmaceutical attributes of various bioactive including curcumin (Saha et al., 2007), hesperetin (Maiti et al., 2009), chrysophanol (Singh et al., 2013) and mangiferin (Telange et al., 2021b). Phospholipids complex are comprised of bioactive and phospholipids. In this study, we utilized Phospholipon^®^90H as drug delivery nanocarriers. Like other phospholipids, the Phospholipon^®^90H also acts as compound lipids and establishes the significant components of the plasma membrane (Kidd, 2009). As part of the compound lipid and the membrane, the Phospholipon^®^90H-based complex can interact with amphiphilic phospholipidic bilayers of the membrane. This interaction further increases the miscibility of the complex within the bilayers, facilitating the transportation of hydrophobic bioactive across the membrane and enhancing its therapeutic efficacy (Renukuntla et al., 2013; Saoji et al., 2016). Phospholipon^®^90H amphiphilic feature can improve the drug water and lipid solubility due to positive and negative charges on it (Constantinides et al., 2008). Supporting this, the phospholipids bearing hydroxyl groups in the presence of an esterification mechanism could develop an intermolecular association with active hydrogen molecules of bioactive. Interaction forms an amphiphilic compound with an increased ability to cross the biological membrane and enhance the bioavailability and therapeutic activity (Bildstein et al., 2011). The mechanism also happened in the genistein-Phospholipon^®^90H complex formation process. The Phospholipon^®^90H could establish the hydrogen bonding with hydroxyl groups of GEN in the presence of methanol, forming amphiphilic genistein – phospholipids complex (GPLC) and enhancing the aqueous solubility, permeability, bioavailability and anti-inflammatory potential of GEN. Therefore, the current research was carried out based on the advantages, benefits, and application of phospholipid complexes.

In this study, we prepared and characterized the phospholipids complex of genistein. The GPLC was developed using the solvent evaporation method. The GPLC complex was physico-chemically characterized using particle size and zeta potential, Fourier transforms infrared spectrophotometry, differential scanning calorimetry, powder x-ray diffractometry, and proton nuclear magnetic resonance. Similarly, the complex was functionally characterized by saturation solubility, in vitro dissolution, ex vivo permeation, and oral bioavailability studies. Additionally, the anti-inflammatory potential of GPLC was preliminarily tested in a carrageenan-induced albino rat model.

## 2. Experimental

### 2.1. Materials

Genistein was obtained from Green Heaven Institute of Management and Research, Nagpur, India. Phospholipon^®^90H was obtained from Lipoid GmbH, Ludwigshafen, Germany. Carrageenan was purchased from Sigma-Aldrich Corporation, St. Louis, MO, USA. Acetonitrile, acetic acid, 1, 4-dioxane, ethyl acetate, n-hexane, and methanol were purchased from Loba Chemicals Pvt. Ltd., Mumbai, India. The procured chemicals were of analytical grade used in this research work.

### 2.2. Methods

#### 2.2.1. Preparation of genistein-Phospholipon^®^90H complex (GPLC)

Genistein-Phospholipon^®^90H complex (GPLC) was prepared using the well-established solvent evaporation method with some modifications (Bhattacharyya et al., 2014). Briefly, the GEN and Phospholipon^®^90H, as per molar ratios of (1:0.5, 1:1, 1:1.5, and 1:2), were accurately weighed and transferred into a 100 mL round bottom flask. The transferred ingredients were mixed with 20 mL of methanol and refluxed at 40 °C for 5 h. Next, the refluxed solution was concentrated into 2–3 mL liquid residue. The residue was treated with 10 mL of n-hexane forming GPLC. The GPLC was vacuum dried at 40 °C for 24 h. The dried complex was sieved (16#) for uniform GPLC particle size. The resulting complex was transferred and preserved into nitrogen-flushed amber-colored glass vials and processed for characterization studies.

#### 2.2.2. Determination of drug content

The GEN content within the prepared GPLC formulations was determined using a UV-visible spectrophotometric method reported earlier (Maryana et al., 2016). Briefly, the GPLC complex (∼5 mg of GEN) was accurately weighed and transferred into a 10 mL volumetric flask. The complex was dissolved in a sufficient amount of methanol. After this, the dissolved sample was diluted appropriately and analyzed for UV absorbance using a UV-visible spectrophotometer (Model: UV-1800, Shimadzu, Japan) at a maximum wavelength of ∼260 nm. Likewise, the Phospholipon^®^90H solution was also prepared with the same method, compared, and analyzed against the sample solution to prevent carrier interference. A below-described Eq. (1) was used to calculate the GEN content within the GPLC formulations.

(1) $$\%\;\mathrm{Drug}\;\mathrm{content}=\;\frac{\mathrm{Absorbance}\;of\;\mathrm{test}}{\mathrm{Absorbance}\;of\;\mathrm{standard}\;}\;\times 100$$

#### 2.2.3. Determination of % entrapment efficiency

A method earlier described by Maryana et al. was employed to estimate the encapsulation efficiency of GEN in prepared GPLC formulations (Maryana et al., 2016). Briefly, an approximate amount of ∼10 mL GPLC formulations was centrifuged at 15000 × g for 1 h at room temperature. After centrifugation, the supernatant was removed using a micropipette, diluted suitably using methanol, and analyzed for GEN content using a UV-visible spectrophotometer (Model: UV-1800, Shimadzu, Japan) at a maximum wavelength of ∼260 nm. The below-described Eq. (2) was employed to calculate the % entrapment efficiency of GEN in GPLC.

(2) $$\%\;\mathrm{Entrapment}\;\mathrm{efficiency}=\frac{\mathrm{Amount}\;of\;\mathrm{encapsulated}\;\mathrm{genistin}}{\mathrm{Amount}\;of\;\mathrm{total}\;\mathrm{genistin}\;}\times 100$$

#### 2.2.4. Particle size and zeta potential analysis

Particle size and zeta potential parameters are often used to investigate nanoparticulate systems dispersed in the aqueous media. The Photon Cross-Correlation Microscopy (PCCS) equipped with dynamic light scattering (DLC) technology was used to determine the particle size distribution of GEN within the GPLC formulations as per the procedure reported earlier in the literature (Telange et al., 2017). Briefly, the GPLC formulations (∼5 mg) were weighed and dispersed in 10 mL of deionized water. The dispersion was placed into a particle size analyzer (Model: Nanophox Sympatec, GmbH, Clausthal-Zellerfeld, Germany) and recorded the particle size within the sensitivity range of ∼1 nm to 10 µm. The obtained results were interpreted using the instrument attached software. Likewise, the GPLC aqueous dispersion was also analyzed for zeta potential analysis. The formulations were placed into the chamber and analyzed for zeta potential within the sensitivity range of ∼ ± 200 mV using Nano Particle Analyzer (Model: NanoPlusTM-2, Particulate System, Norcross, GA, USA) with DLS technology. The analysis was done at room temperature.

#### 2.2.5. Fourier transform-infrared spectroscopy (FT-IR)

FT-IR technique is used to identify the occurrence of different functional groups present in formulation components. The samples of pure GEN, phospholipon^®^90H, physical mixture (PM) of GEN and Phospholipon^®^90H and GPLC formulations were analyzed for their functional group interaction using FT-IR spectrophotometer (Model: IRA-IS WL, Shimadzu, Kyoto, Japan). Briefly, the samples and potassium bromide (KBr, FT-IR grade) powder were mixed using agate mortar and pestle. The powder was compressed into thin transparent disc and scanned between 4000 − 400 cm^−1^. The scanned images were read and interpreted using instrument-attached software (Control Software, Version 1.10). The method reported earlier in the literature was employed to prepare and analyze FT-IR samples (Telange et al., 2019).

#### 2.2.6. Differential scanning calorimetry (DSC)

DSC is used to determine the thermal changes in the physico-chemical properties of the samples as a function of time and temperature. The samples of pure GEN, Phospholipon ^®^90H, and GPLC formulations were analyzed for their thermal changes as a function of time and temperature using a differential scanning calorimeter (Model: DSC-1821e, Mettler Toledo AG, Analytical, Schwerzenbach, Switzerland). Briefly, the sample (∼2 mg) was accurately weighed and transferred into the analysis chamber of the instrument. Before analysis, the DSC instrument was nitrogen purged and calibrated using high-purity standard Indium. After this, the samples were heated at a heating rate of 10 °C/min within a heating range of 40 °C to 400 °C. The DSC scanned images were read and interpreted using instrument-attached software (UA, V4.5A, build 4.5.0.5). The method reported by our group earlier was used for the DSC analysis (Telange et al., 2021a).

#### 2.2.7. Powder x-ray diffractometer (PXRD)

PXRD technique determines the formulation component crystallinity degree and amorphous nature. A PXRD instrument (Model: D8 ADVANCE, Bruker AXS, Inc., Madison, WI, USA) was utilized in this study. The crystalline characteristics of pure GEN, Phospholipon^®^90H, and prepared GPLC formulations were analyzed using the PXRD instrument. Briefly, an approximate amount (∼50 mg) of samples was loaded in the analysis area and irradiated using a CuKβ radiation source (ƛ = 1.5406 A^0^). The diffraction signal generated from samples was picked up and detected using a silicon strip-based detector ((LYNXEYE^TM^). Following this, the developed spectrum of each sample was analyzed and interpreted using software attached instrument. Our group reported a procedure earlier in the literature followed in sample analysis (Dave et al., 2018).

#### 2.2.8. Proton nuclear magnetic resonance (^1^H-NMR)

The ^1^H-NMR technique is employed to characterize the carbon-hydrogen relationships between the API and formulations. Pure GEN and GPLC formulations samples were analyzed using 400 MHz FT-NMR spectrophotometers (Model: Bruker Advance II, Bruker BioSpin, Billerica, USA).

#### 2.2.9. Saturation solubility analysis

A method earlier reported by Saoji et al. was used to determine the solubility of pure GEN or prepared GPLC formulations in water (Saoji et al., 2016). Briefly, an excess amount of pure GEN or GPLC formulations was weighed and mixed with 5 mL of water in sealed glass containers resulting in the formation of dispersion. The dispersion was agitated using a rotary shaker (Model: RS-24 BL, REMI Laboratory Instruments, Remi House, Mumbai, India) at 37 °C for 24 h. Next, the dispersion was centrifuged at 1500 RPM for 25 min at 37 °C. Following centrifugation, the obtained supernatant was collected and filtered using a membrane filter (0.45 µ). From this filtrate, 1 mL solution was removed, diluted suitably, and analyzed for absorbance using a UV-visible spectrophotometer (Model: UV-1800, Shimadzu, Kyoto, Japan) at a maximum wavelength of ∼294 nm.

#### 2.2.10. In vitro dissolution studies

The comparative in vitro dissolution performance of pure GEN or prepared GPLC formulations was carried out in phosphate buffer (0.05 M, pH 6.8) using USP Type II dissolution tests apparatus (Model: TDT-08LX, Elecctrolab India Pvt. Ltd., Mumbai, India). The procedure described by Dhore et al. was employed to study the dissolution of pure drug or formulations (Dhore et al., 2017). Briefly, the pure GEN (∼50 mg) or prepared GPLC formulations (∼50 mg of GEN) were accurately weighed and dispersed in 500 mL phosphate buffer dissolution media. The dispersed contents were continuously stirred at a speed of 50 RPM, and the media temperature was maintained at 37 ± 0.5 °C throughout the studies. Next, the samples were removed at fixed intervals and compensated using the same volume of dissolution media. Samples were appropriately diluted using phosphate buffer and analyzed for GEN absorbance using a UV-visible spectrophotometer (Model: UV-1800, Shimadzu, Kyoto, Japan) at a maximum wavelength of ∼294 nm. The computed absorbance values were calculated and reported in percentage cumulative GEN release.

#### 2.2.11. Ex vivo permeability studies

The comparative ex vivo permeation of pure GEN and GPLC formulations across the intestinal membrane was performed in phosphate buffer (0.05 M, pH 6.8). The pure GEN (∼10 mg) or GPLC (∼10 mg of GEN) was injected with a blunt needle into six intestinal membrane. A thread was used to bind the intestine membrane two sides together firmly. Each membrane was held in a flask containing 10 mL of Ringer solution. The complete system was kept at 37 ± 1 °C in a water bath shaken at 100 RPM and aerated using a lab aerator. The samples were taken from outside the membrane after 15 mins for 2 h, and the medium was changed entirely with the new medium. HPLC was used to evaluate the samples. The Waters HPLC system installed the reverse-phase Phenomenex C18 analytical column (150 mm × 4.6 mm). A mobile phase of acetonitrile: water: glacial acetic acid (25:68:7 v/v) was pumped at a flow rate of 1.0 mL/min. The injection volume was 20 µL, and the effluent was monitored at ∼263 nm.

#### 2.2.12. In vivo anti-inflammatory activity

The in vivo anti-inflammatory activity of the prepared GPLC formulation was determined using carrageenan-induced paw edema in rats. Male and female Wistar rats were randomly divided into six groups. Group I **(**vehicle control) animals received a subplantar injection of carrageenan and were treated with 0.1% water. Group II animals received a subplantar injection of carrageenan and were treated with diclofenac (10 mg/kg). Group III animals received a subplantar injection of carrageenan and were treated with GPLC (5 mg/kg). Group IV animals received a subplantar injection of carrageenan and were treated with GPLC (∼10 mg/kg). Group V animals received a subplantar injection of carrageenan and were treated with GPLC (∼20 mg/kg).

Animals used in experiments received the medication or vehicle orally an hour before receiving an injection of carrageenan. Each rat right hind paw received a subplantar injection of 0.1 mL of freshly made carrageenan suspension at 1% v/v in distilled water. The paw volume was measured using a plethysmometer before (0 hours) and at intervals of 1, 2, 3, 4, and 5 h following carrageenan injection. The proportion of edema inhibition was then determined.

#### 2.2.13. Oral bioavailability studies

Albino Wister rats weighing 300–350 g were separated into two groups for in vivo pharmacokinetic investigations. Pure GEN (50 mg/kg, p.o.) and GPLC formulations (∼50 mg/kg, p.o.) were given orally to groups I and II, respectively. At 2, 4, 8, 16, and 32 h following oral delivery, the blood samples (0.5 mL) were obtained and put into the Eppendorf tubes.

#### 2.2.14. HPLC method development

The amount of GEN isolated from plasma was measured using a C18 ODS column (5 m, 4.6 mm × 250 mm) and a reversed-phase HPLC system with a PDA detector (Model: Shimadzu, Japan). The ratio of acetonitrile: water: glacial acetic acid in the mobile phase solution was 55:45:2% v/v. The detecting wavelength was ∼280 nm, and the flow rate was adjusted to ∼1.0 mL/min. GEN and emodin had roughly 6.8 and 10.8 min retention durations, respectively. The range of linearity was ∼5.5 to 440 ng/mL. For intra and interday testing, the coefficient of variance was less than 10%. GEN was recovered from isolated plasma solutions at an average rate of more than 90%.

#### 2.2.15. Extraction of genistein from plasma and sample preparation

The blood samples were centrifuged at 5000 RPM for 10 min at 4 °C to separate the plasma. 200 µL of plasma were mixed with a 2.5 µL internal standard solution that contained 2.45 µg/mL of emodin dissolved in methanol. GEN was extracted by combining diluted plasma with 500 µL of ethyl acetate and vertexing the mixture for 3 min. The samples were centrifuged at 10,000 RPM for 10 min to separate the supernatants. The extraction process was repeated by adding 500 µL ethyl acetate to the leftover material. The two supernatants were mixed and dried with a stream of nitrogen gas. The remaining materials were centrifuged at 10,000 RPM for 10 min after being resuspended in 100 µL of methanol. After that, 20 µL of the supernatants were used for HPLC analysis.

#### 2.2.16. Study of pharmacokinetic parameters

The pharmacokinetic software (KINETICA 5.0) was used to calculate the pharmacokinetic parameters. The mean and standard error of the mean was used to represent the pharmacokinetic data. The one-way ANOVA test was used to examine the group differences.

#### 2.2.17. Statistical analysis

The drug content, entrapment efficiency, and solubility studies results are presented as mean ± standard deviation. Likewise, the in vitro dissolution, ex vivo permeation, oral bioavailability, and in vivo anti-inflammatory activity results are shown as mean ± standard error of the mean. The one-way ANOVA test was carried out to study the difference between animal groups, followed by Dunnett’s test. The obtained *p*-value of less than 0.05 was considered a significant one.

## 3. Results and discussion

### 3.1. Preparation of GPLC

A Phospholipon^®^90H-based complex of GEN was created in this study using the solvent evaporation approach to enhance its water solubility and physiological transport. Based on several studies showed that isoflavonoids are hydrophobic, resulting in lower water solubility (Karthivashan et al., 2016; Telange et al., 2016; Yue et al., 2010). These investigations suggested that a phospholipid-based complex was prepared to utilize dichloromethane, tetrahydrofuran, or 1, 4-dioxane as the solvent of choice. However, the preliminary study revealed that GEN was poorly soluble and precipitated from DCM, THF, and 1, 4-dioxane. Alternative solvents were therefore investigated to address this issue. Methanol was selected as the preferred solvent for the formulation of GPLC because it is semi-polar with a low dielectric constant. Apart from this, we also used n-hexane as a nonsolvent, which reduced the complex solubility and increase its precipitation from the solution. The obtained complex acquired lipophilic character and thus, showed higher solubility in non-polar and aprotic solvents, where GEN and Phospholipon^®^90H showed insolubility.

The GPLC was developed using the methanol-based solvent evaporation method. In the presence of methanol, the hydrophilic polar head of Phospholipon^®^90H interacted via hydrogen bonding interaction with polar functional groups of GEN. This interaction facilitates the incorporation of the GEN molecule within the polar head of Phospholipon^®^90H, whereas, the hydrophobic tail part of this phospholipid provides the correct orientation to this complex resulting in the formation of an amphiphilic complex with improved transportation from the aqueous environment to the lipid soluble environment (Hou et al., 2013).

### 3.2. Determination of drug content and entrapment efficiency

The drug content and entrapment efficiency of GEN within the GPLC formulations were found to be ∼88.54 ± 0.82% w/w and ∼97.88 ± 0.36% w/w, respectively. The higher content and entrapment efficiency were likely attributed to the chemical interaction between GEN and Phospholipon^®^90H. Moreover, the selected amount of Phospholipon^®^90H could entrap GEN successfully, resulting in higher entrapment efficiency.

### 3.3. Particle size and zeta potential analysis

**Figure 1.**
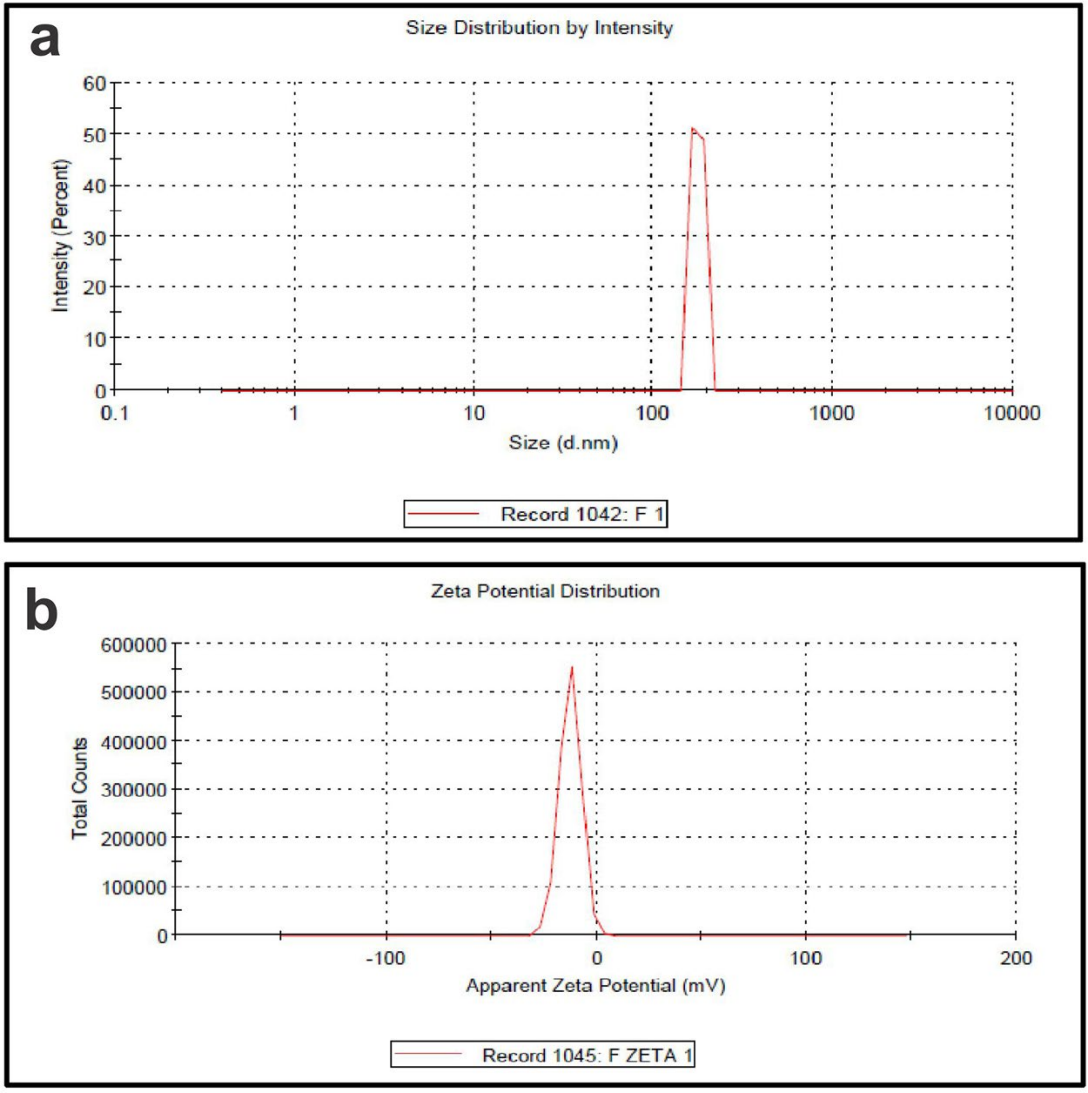
(a) Particle size and (b) zeta potential analysis of GPLC formulations.

Particle size and zeta potential are excellent indicators of nanoparticle physical stability. The evodiamine-phospholipid complex particle size of ∼246.1 nm significantly improved the drug sustained release and oral absorption efficiency (Zhang et al., 2012). The transport of bigger particles (larger than 5 mm) may include lymphatics, whereas the transport of smaller particles (less than 500 nm) may involve endocytosis (Lefevre et al., 1978; Savić et al., 2003). The particle size analysis of GPLC formulation (Figure 1a) revealed around ∼176.9 nm, with polydispersity indices of 0.28 (< 3) shows its suitable for oral drug delivery. The low polydispersity index indicates a limited particle size distribution range within complex formulations. Colloidal dispersion stability is evaluated by its zeta potential. Previous literature has suggested that a zeta potential value of ± 10 mV provides considerable stability to the system (Mazumder et al., 2016). The zeta potential for the GPLC formulation (Figure 1b) was found to be ∼− 6.78 mV, respectively. The obtained value appeared close to ∼− 10 mV, indicating the physical stability of GPLC formulations. The zeta potential value will vary depending on the kind and composition of phospholipids. The lower zeta potential of the complex can be explained by the contribution of Phospholipon^®^90H to the formation of negative charges in an aqueous environment with a neutral pH value. Therefore, good physical stability for GPLC formulations was suggested by reduced particle size, decreased polydispersity index, and modest zeta potential value.

### 3.4. Fourier transform infrared spectrophotometry

**Figure 2.**
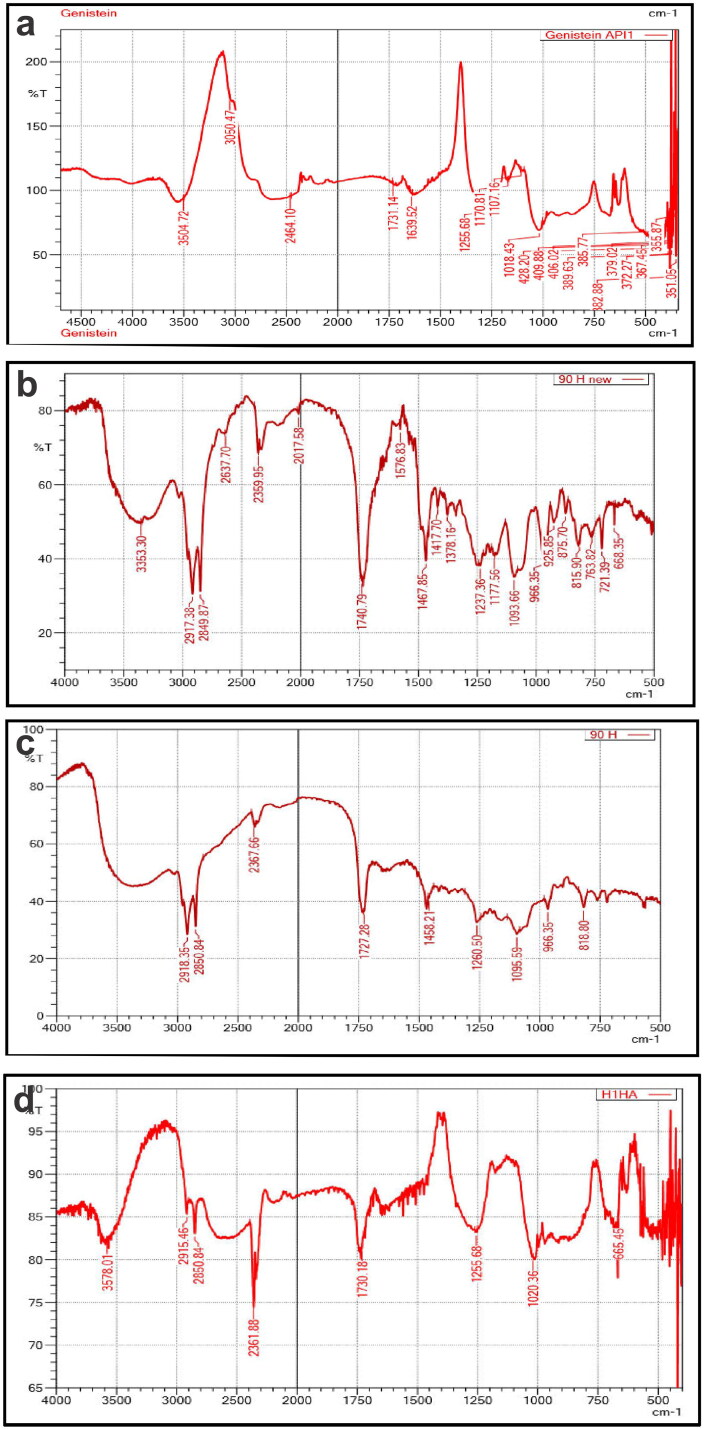
Fourier transforms infrared spectrophotometry of (a) pure GEN, (b) Phospholipon^®^90H, (c) PM of pure GEN and Phospholipon^®^90H, and (d) GPLC formulations.

The FT-IR spectrum of pure GEN, Phospholipon^®^90H, Physical mixture (PM) of pure GEN, and Phospholipon^®^90H, and GPLC formulations are shown in Figure 2 (a, b, c and d). Pure GEN (Figure 2a) shows absorption peaks at ∼3504.72 cm-1 for O-H stretching and N-H stretching, 1731.14 and 1639.52 cm-1 for C = O stretching, 1639.52 cm-1 for C = C stretching, and 1255.68 for C-O stretching (Pandit and Patravale, 2011) (Figure 2b) displays the FT-IR spectrum of Phospholipon^®^90H. It exhibits the absorption peaks at the position of ∼3353.30 cm-1 for O-H stretching, 2917.38 and 2849.87 cm-1 for C-H stretching, 1740.79 cm-1 for C = O stretching in the fatty ester, 1237.36 cm-1 for P = O stretching, 1093.66 cm-1 for P-O-C stretching and 966.35 cm-1 for (-N_+_(CH_2_)_3_). The results are consistent with earlier published reports ((Murugan et al., 2009). The PM FT-IR spectrum (Figure 2c) shows the additive peaks of ∼2850.84 cm-1, 1727.28 cm-1, and 1095.59 cm-1 corresponding to pure GEN and Phospholipon^®^90H. Finally, the FT-IR spectrum of GPLC formulations is shown in (Figure 2d). It showed peaks at ∼3578.01 cm-1, 2361.88 cm-1, 1730.18 cm-1, 1255.68 cm-1, 1020.36 cm-1 and 665.45 cm-1. The appearance and shifting of these peaks compared to the original indicate that hydrogen bonding, ion-dipole forces and van der Waal forces were established between pure GEN and Phospholipon^®^90H forming GPLC.

### 3.5. Differential scanning calorimetry

**Figure 3.**
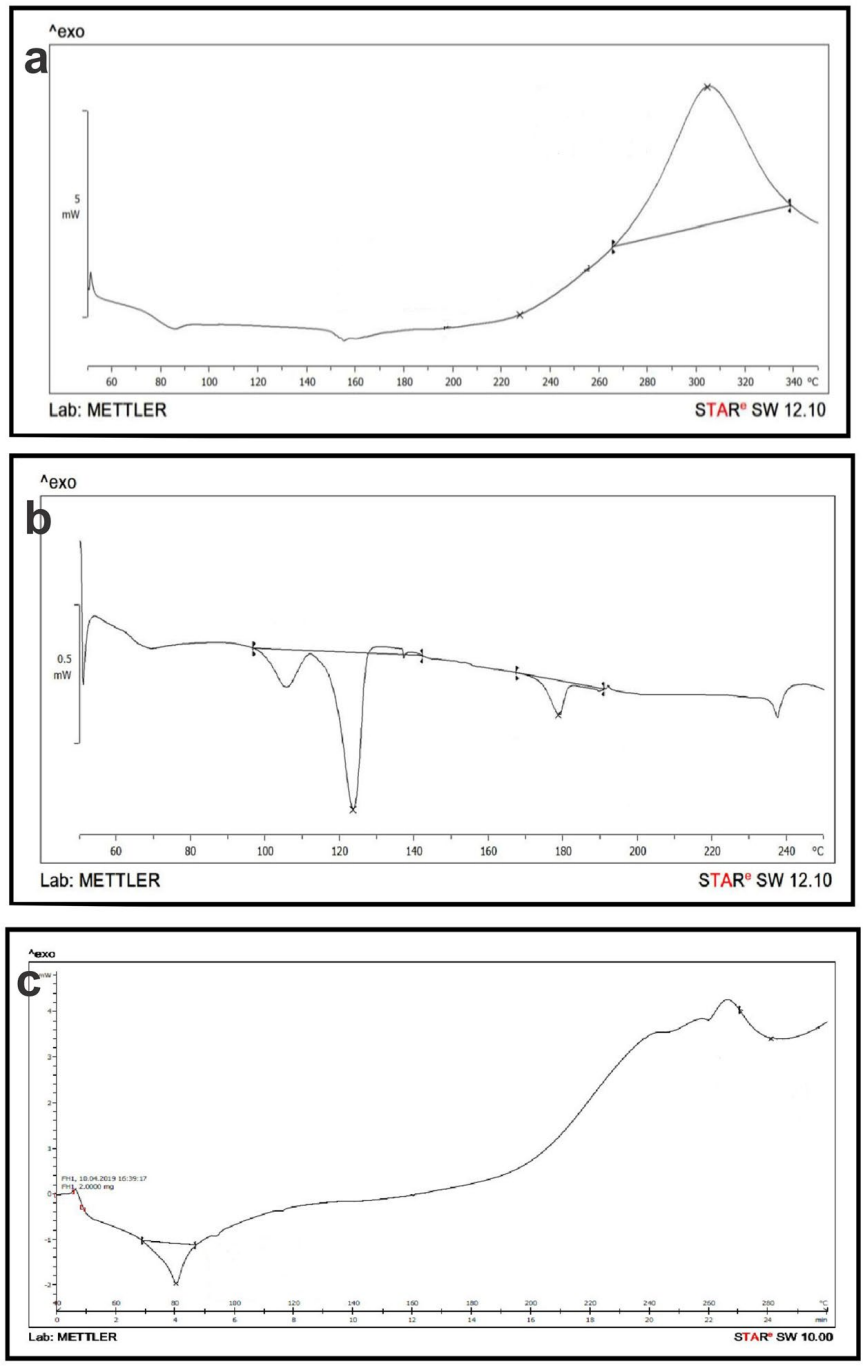
Differential scanning calorimetry analysis of (a) pure GEN, (b) Phospholipon^®^90H, and (c) GPLC formulations.

The thermograms of pure GEN, Phospholipon^®^90H, and GPLC formulations are shown in Figure 3 (a, b and c). Pure GEN (Figure 3a) shows a peak at ∼304.77 °C, indicating its crystalline nature. Phospholipon^®^90H thermograms (Figure 3b) revealed two separate peaks, with the first peak having a lesser intensity at ∼123.46 °C and the second peak having a comparatively sharper peak at ∼178.64 °C. The second peak shape denotes a phase-transition point, while the first peak describes the strength attributed to melting. It is suggestive of a transition from a gel state to a liquid crystalline form, along with isomeric modifications to the phospholipid hydrocarbon segment, which are induced by movements of the molecules polar component as temperature rises. The thermograms of GPLC (Figure 3c) showed a sharp new peak at ∼80.27 °C with a complete absence of original peaks of GEN and Phospholipon^®^90H. It suggests that weak intermolecular forces (hydrogen bonds and van der Waals interactions, etc.) between GEN and Phospholipon^®^90H could develop, resulting in the formation of GPLC.

### 3.6. Powder x-ray diffractometry

**Figure 4.**
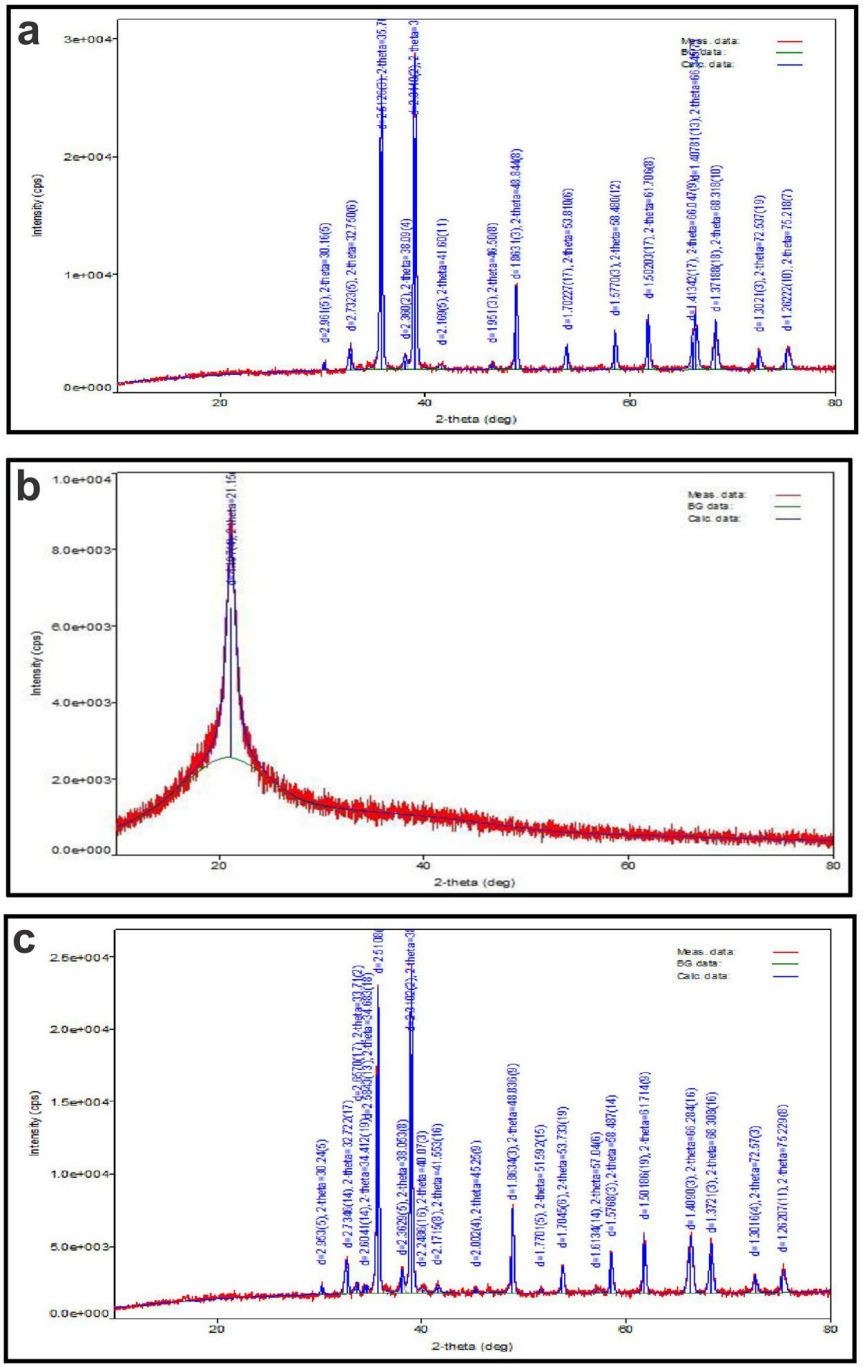
Powder x-ray diffractometry of (a) pure GEN, (b) Phospholipon^®^90H, and (c) GPLC formulations.

Figure 4(a, b and c), display the diffractograms of pure GEN, Phospholipon^®^90H, and prepared GPLC formulations. Pure GEN (Figure 4a) exhibits diffraction peaks at ∼6.4°, 12.7°, 18.4°, 25°, 26.36°, 28.6°^,^ and 29.4°, respectively, indicating its crystalline nature (Zafar et al., 2021). Phospholipon^®^90H (Figure 4b) revealed a single diffraction peak at a position of ∼21.15° indicating its partial amorphous nature. The GPLC formulations (Figure 4c) displayed slightly lower intensity diffraction peaks than the original one because of the contribution of Phospholipon^®^90H. It suggests that GEN could be molecularly dispersed within the Phospholipon^®^90H matrix, resulting in complex formation with lower intensity peaks.

### 3.7. Proton nuclear magnetic resonance

**Figure 5.**
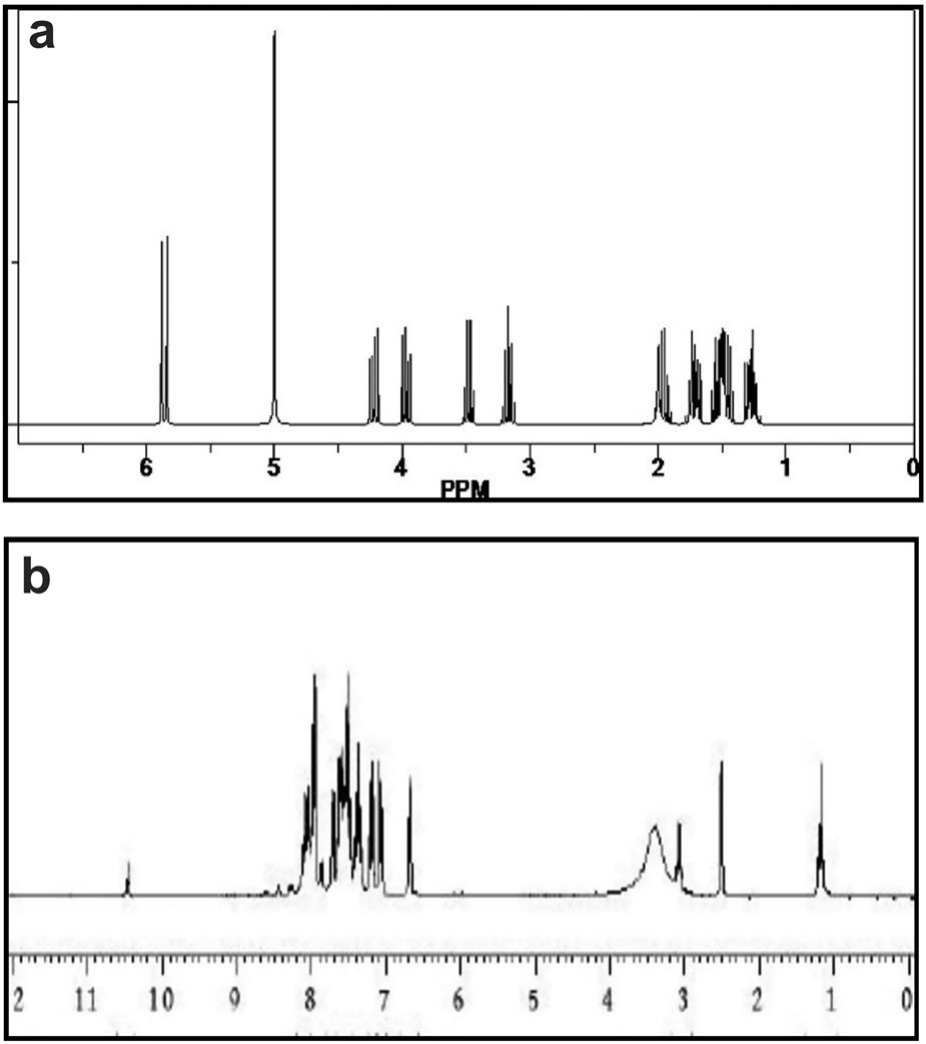
Proton nuclear magnetic resonance analysis of (a) pure GEN and (b) GPLC formulations.

The ^1^H-NMR spectra of pure GEN and GPLC formulations are shown in Figure 5(a and b), respectively. Pure GEN ^1^H-NMR spectrum (Figure 5a) measured in d6-DMSO represents the presence of phenyl protons in the isoflavone skeleton. The peaks at the ∼δ 6.21 (1H, s, H-6) and δ 6.33 (1H, s, H-8) indicate a chemical shift toward the downfield region. Compared to pure GEN, the peak of GPLC (Figure 5b) at ∼δ 1.24 showed a lower intensity peak, and broad signals between the region of ∼δ 6.5 to δ 8.2 accounts for exchanging of protons with the Phospholipon^®^90H. This phenomenon suggests that weak interaction forces could occur between the GEN and Phospholipon^®^90H, resulting in the development of GPLC.

### 3.8. Saturation solubility analysis

The comparative aqueous solubility analysis of pure GEN and prepared GPLC formulations are shown in Table 1. The table shows pure GEN exhibits aqueous solubility around ∼76.58 µg/mL. This lowered solubility could be attributed to GEN lipophilic nature, which closely agrees with earlier published literature (Yang et al., 2012). In contrast to GEN solubility, the GPLC formulations displayed higher aqueous solubility around ∼173.90 µg/mL, indicating that the amorphous nature of the complex could produce higher (∼2-fold) aqueous solubility. Results explained that while complexation, the lipophilic GEN compound may be closely associated with amphiphilic Phospholipon^®^90H in the presence of methanol. The association could facilitate GEN dispersion and partial amorphization by Phospholipon^®^90H, thereby enhancing GPLC aqueous solubility.

**Table 1.** Saturation solubility of pure genistein and prepared GPLC formulations..

Components	Aqueous solubility (µg/mL)
GEN	76.58 ± 2.72
GPLC	173.90 ± 2.32

### 3.9. In vitro dissolution studies

**Figure 6.**
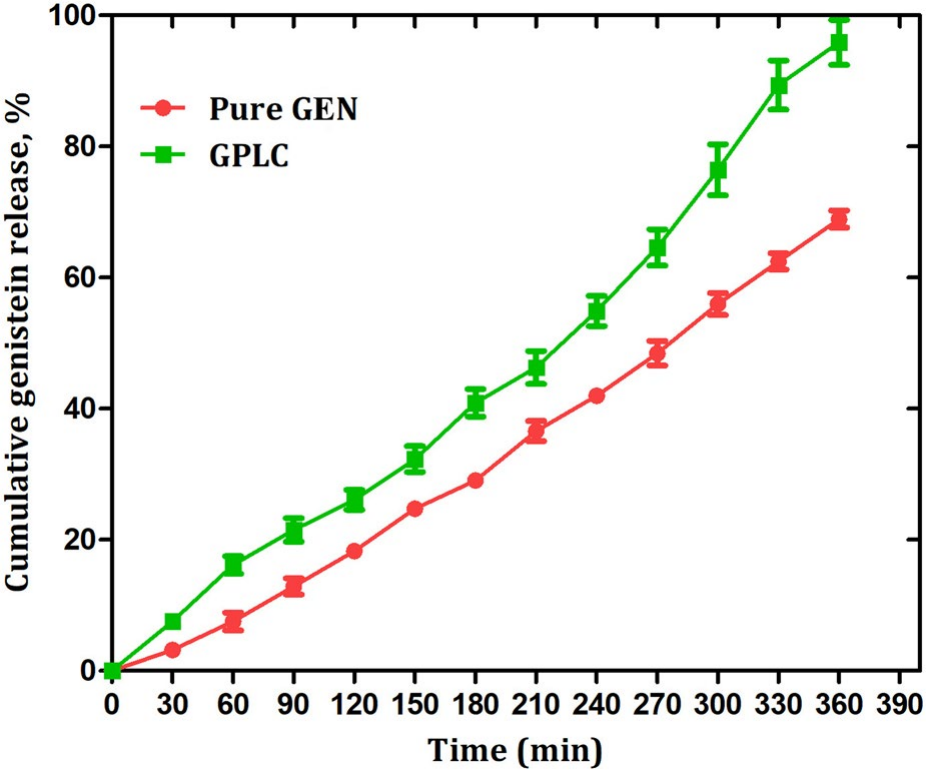
In vitro dissolution release of pure GEN and GPLC formulations. Values are represented as mean ± Std. Dev. (*n* = 3).

Figure 6 describes the comparative in vitro dissolution profile of pure GEN and prepared GPLC formulations in phosphate buffer. The GEN and GPLC formulations displayed parallel dissolution patterns up to 2 h; after that, the GPLC formulations enhanced the rate and extent of GEN dissolution. The pure GEN showed only ∼69% of dissolution at the end of 6 h. The GPLC formulations at the end of 3 h displayed around ∼41% dissolution; after that, the same formulation enhanced the GEN dissolution by ∼96% at the end of 6 h, indicating the amorphous nature of GPLC improves the rate and extent of GEN dissolution. Moreover, the enhanced GPLC dissolution could be attributed to the increasing wettability and solubility of amorphized GPLC particles in the dissolution media leading to enhance the complex solubility (Perrut et al., 2005). It also suggests that the conversion of crystalline GEN to an amorphous form due to the complexation phenomenon improve the rate and extent of drug dissolution in phosphate buffer. According to the dissolution study, it was also observed that the GPLC showed dual release behavior i.e. burst and sustained release. The initial burst release of around ∼41% could be attributed to the adsorption of GEN on the complex surface which could exhibit dissociation from the complex and then release into the dissolution media. The sustained release of around ∼96% could be attributed to the diffusion of dissociated GEN from the phospholipids suggesting that GPLC is stable and thus, showed prolonged release compared to pure GEN. The release data of the GPLC formulations were analyzed using kinetic models such as zero order, first order and Higuchi model, respectively. Following analysis, the zero order, first order and Higuchi models show the correlation coefficient values around (*R^2^* = 0.9025), (*R^2^* = 0.9384) and (*R^2^* = 0.9564), respectively. Compared to all, the higher Higuchi model value indicating diffusion could be the mechanism for the release of GEN from GPLC formulations. Moreover, the Korsmeyer-Peppas model analyzed release exponent value (*n*) around ∼0.48 also suggesting that Fickian diffusion is the suitable dissolution mechanism for the dissolution of GEN from optimized GPLC formulations. This diffusion mechanism was described in two steps. First, the GEN compound dissociates from the complex; second, the dissociated GEN diffuses out from the phospholipid matrix. This two-step diffusion mechanism could be accountable for significantly enhancing the GEN dissolution rate.

### 3.10. Ex vivo permeation studies

**Figure 7.**
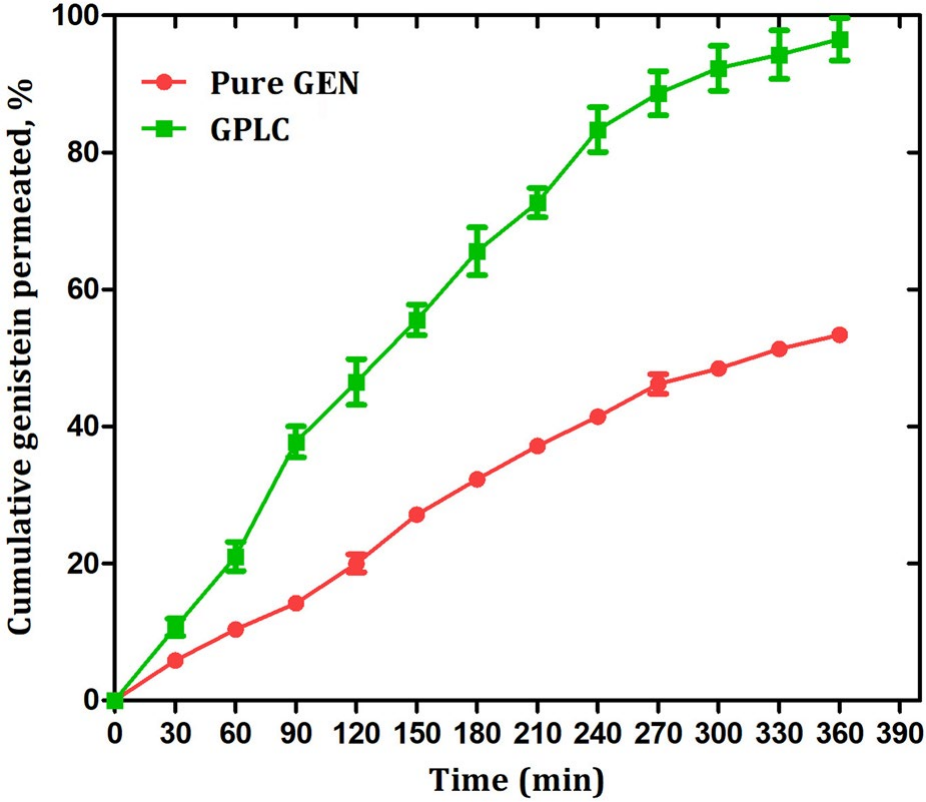
Ex vivo permeation profile of pure GEN and GPLC formulations. Values are represented as mean ± Std. Dev. (*n* = 3).

Figure 7 shows the comparative ex vivo permeation performance of pure GEN and GPLC complex. At the end of six hours, the pure GEN showed only ∼53% of drug permeation across the intestinal membrane, indicating that GEN has a poor permeation profile (Rothwell et al., 2005). The GPLC formulations enhanced the rate and extent of GEN permeation across the intestinal membrane compared to pure GEN. The complex displayed ∼96% of GEN permeation, indicating the complex adopted amorphous nature because Phospholipon^®^90H which could increase the wettability and solubility of the complex, thereby enhancing its permeability. It also suggests that amphiphilic phospholipids complex interact with amphiphilic phospholipids bilayers of the intestinal membrane. This interaction increases the miscibility of the complex within the membrane, increasing the permeation of GEN across the membrane (Saoji et al., 2016). Additionally, the amphiphilic GPLC affinity toward the membrane causes significant destabilization of the membrane. This further creates the pore within the membrane and provides easy permeation to the complex through the exchange of coating lipids in between the GPLC and phospholipids bilayer of the membrane. The partial and complete wrapping in support of GPLC permeation via pore channel also suggests the enhanced complex permeation (Guo et al., 2016; Contini et al., 2018). Moreover, the Phospholipon^®^90H is a class of phospholipids which shows biocompatible and biodegradable advantages similar to intestinal membrane phospholipids. This advantage also recognizes the Phospholipon^®^90H as a smart carrier which could facilitate drug transport across the intestinal membrane thus, enhancing complex permeability.

### 3.11. In vivo anti-inflammatory activity

Carrageenan is an inflammatory inducer. It produced inflammation via vasodilation and tumor necrosis factor (TNF – α) liberation. The comparative in vivo anti-inflammatory potential of diclofenac at a dose of (∼10 mg/kg) and GPLC formulations at a dose level of ∼(5 mg/kg, 10 mg/kg, and 20 mg/kg) in carrageenan-induced albino rat model are discussed below. The carrageenan (group I animals) at a dose of (∼10 mg/kg) enhanced the paw volume and continued to increase up to 5 h. Compared to this, the diclofenac (group II animals) at a dose of (∼10 mg/kg) inhibited a significant amount of paw edema around ∼37.32, 46.08, 51.15, 56.59, and 67.10 for a period of 1 to 5 h, respectively. The GPLC formulations at a dose of (∼20 mg/kg) more significantly inhibited the paw edema around ∼41.58, 45.70, 52.93, 57.60 and 73.65 for a period of 1 to 5 h, respectively, compared to the same formulation at a dose level of (∼5 mg/kg, group III animals) and dose level of (∼10 mg/kg, group IV animals). Findings indicate that complex at higher doses remarkably inhibited paw edema and could be attributed to enhanced solubility, dissolution, permeation, diffusion, and complex bioavailability.

### 3.12. Oral bioavailability studies

**Figure 8.**
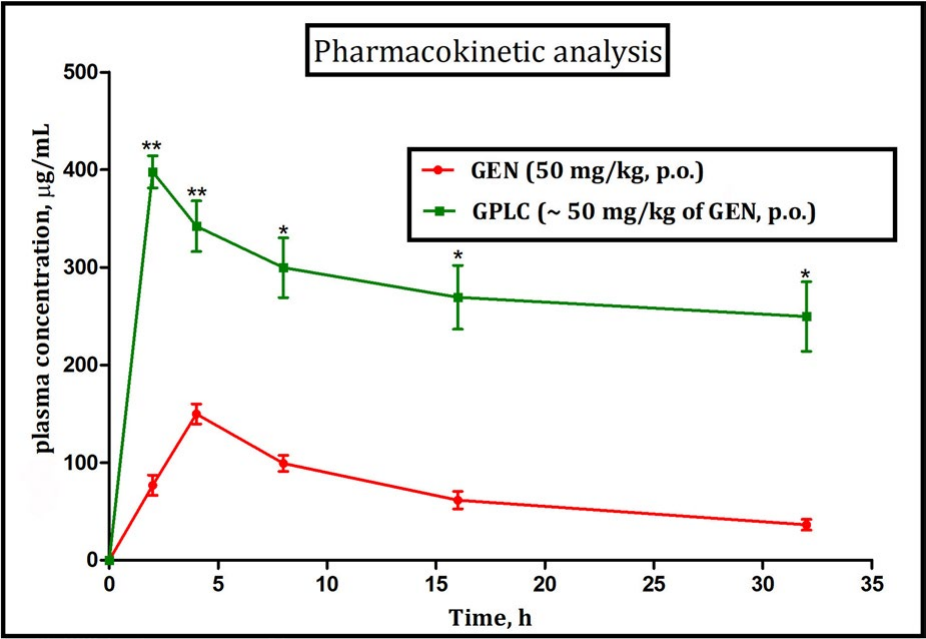
Mean plasma concentration-time profile curve following oral administration of GEN (50 mg/kg, p.o.), and GPLC (∼50 mg/kg of GEN, p.o.). Values are mean ± SEM (*n* = 6). *p < 0.05 and **p < 0.01 (significant with respect to GEN treated group).

The oral bioavailability studies, i.e. mean plasma concentration vs time profile curve of pure GEN and GPLC formulations at a dose level of (∼50 mg/kg, p.o.) administered to group I and II animals, and their results are shown in Figure 8. As shown in the figure, the GPLC formulation enhanced the Cmax value (∼343.88 µg/mL-1) as compared to lower Cmax value (∼149.99 µg/mL) observed in pure GEN. Likewise, the complex lowered the tmax value around (∼2.2 h) compared to a higher value (∼4.43 h) in pure GEN, indicating that the complex provides the sustained and controlled delivery of GEN. Statistical software (KINETICA Version 5.0) also estimated some additional pharmacokinetic parameters, and their results are shown in Table 2. The GPLC formulations significantly enhanced the AUC_0-t_ value (∼8013.24 µg/mL-1h) compared to pure GEN lower value (∼3811.31 µg/mL^−1^h), suggesting enhanced bioavailability of GEN. The complex also improved the MRT value (∼184.43 h), compared to pure GEN lowered the value (∼17.38 h), indicating better availability of complex within the body following oral administration. The complex improved the elimination half-life of the GEN around (∼11.99 h) compared to pure GEN value (∼3.6 h), suggesting amphiphilic complex enhanced the circulation time within the blood circulation and showed sustained release behavior. This enhancement could be explained by the fact that while complexation, the amphiphilic Phospholipon^®^90H facilitates the dispersion, and partial amorphization of the GEN molecule increase its solubility, dissolution, and permeation. This combined effect could be responsible for the enhancement of the oral bioavailability of complex formulations. Moreover, the prevention of complex first-pass metabolism because of Phospholipon^®^90H inclusion could also find the reason for enhancing oral bioavailability of complex compared to pure GEN. Apart from this, the sustained release of dissociated GEN from the phospholipids matrix barrier may also increase its adhesion, interaction and miscibility with the amphiphilic membrane resulting in the enhancement of GPLC bioavailability.

**Table 2.** Results of pharmacokinetic parameters obtained in group of animals following oral administration of GEN (∼50 mg/kg, p.o.) and GPLC (∼50 mg/kg, p.o.).

Pharmacokinetic parameters	Formulations	
	GEN	GPLC
*C_max_* (µg/mL)	149.99 ± 1.25	343.88 ± 1.65
*T_max_* (h)	4.43 ± 1.10	2.2 ± 1.05
*t*_½el_ (h)	3.6 ± 2.10	11.99 ± 1.14
*AUC_0-t_* (µg h mL^-1^)	3811.31 ± 2.28	8013.24 ± 1.52
*MRT* (h)	17.38 ± 1.36	184.43 ± 0.94

## 4. Conclusions

GPLC was prepared successfully using the methanol-based solvent evaporation method. The optimized GPLC displayed higher entrapment efficiency. The characterization parameters, i.e. FT-IR, DSC, PXRD, and ^1^H-NMR, supported the development of a complex with the formation of hydrogen bonding between GEN and Phospholipon^®^90H. The saturation solubility studies indicated that complex enhanced the GEN aqueous solubility by 2-fold via a partial amorphization mechanism. Results of in vitro dissolution studies suggested that complex improved the GEN dissolution compared to pure GEN. Likewise, the ex vivo permeation studies revealed that the complex significantly enhanced the permeation across the membrane compared to pure GEN. The in vivo anti-inflammatory potential of the complex at a dose of (∼20 mg/kg, p.o.) drastically inhibited the paw edema in the carrageenan-induced albino rat model up to 5 h compared to pure GEN and diclofenac. Pharmacokinetic analysis suggested that complex at a dose of (∼50 mg/kg, p.o.) enhanced the GEN oral bioavailability via enhancing Cmax, tmax, AUC, half-life and mean residence parameters as compared to pure GEN. Findings conclude that phospholipon^®^90H complex nanocarrier successfully modifies the physico-chemical properties of GEN. Therefore, it could be employed as a promising strategy to enhance the biopharmaceutical attributes of bioactive with poor solubility, permeability, and oral bioavailability.
